# Self-organizing behaviors of cardiovascular cells on synthetic nanofiber scaffolds

**DOI:** 10.1063/5.0172423

**Published:** 2023-12-01

**Authors:** Michael M. Peters, Jackson K. Brister, Edward M. Tang, Felita W. Zhang, Veronica M. Lucian, Paul D. Trackey, Zachary Bone, John F. Zimmerman, Qianru Jin, F. John Burpo, Kevin Kit Parker

**Affiliations:** 1 Disease Biophysics Group, Harvard John A. Paulson School of Engineering and Applied Sciences, Harvard University, Boston, Massachusetts 02134, USA; 2 Department of Chemistry and Life Science, United States Military Academy, West Point, New York 10996, USA

## Abstract

In tissues and organs, the extracellular matrix (ECM) helps maintain inter- and intracellular architectures that sustain the structure–function relationships defining physiological homeostasis. Combining fiber scaffolds and cells to form engineered tissues is a means of replicating these relationships. Engineered tissues' fiber scaffolds are designed to mimic the topology and chemical composition of the ECM network. Here, we asked how cells found in the heart compare in their propensity to align their cytoskeleton and self-organize in response to topological cues in fibrous scaffolds. We studied cardiomyocytes, valvular interstitial cells, and vascular endothelial cells as they adapted their inter- and intracellular architectures to the extracellular space. We used focused rotary jet spinning to manufacture aligned fibrous scaffolds to mimic the length scale and three-dimensional (3D) nature of the native ECM in the muscular, valvular, and vascular tissues of the heart. The representative cardiovascular cell types were seeded onto fiber scaffolds and infiltrated the fibrous network. We measured different cell types' propensity for cytoskeletal alignment in response to fiber scaffolds with differing levels of anisotropy. The results indicated that valvular interstitial cells on moderately anisotropic substrates have a higher propensity for cytoskeletal alignment than cardiomyocytes and vascular endothelial cells. However, all cell types displayed similar levels of alignment on more extreme (isotropic and highly anisotropic) fiber scaffold organizations. These data suggest that in the hierarchy of signals that dictate the spatiotemporal organization of a tissue, geometric cues within the ECM and cellular networks may homogenize behaviors across cell populations and demographics.

## INTRODUCTION

Differences in inter- and intracellular structural organization in the various tissues of the heart lead to fundamental differences in tissue formation and arrangement. Cardiovascular tissues are organized spatially to regulate contractile forces (cardiac muscle cells),^1,2^ the endured strain of cyclic loading (valvular interstitial cells),^3^ and the effect of shear stresses subjected to blood flow (vascular endothelial cells).^4^ For example, cardiomyocytes are polarized striated muscle cells that couple at their longitudinal ends, forming specialized cell junctions called intercalated disks.^5,6^ Cardiomyocytes' actin cytoskeleton is organized predominantly within laterally coupled sarcomeres which are serially aligned within the myofibrils that are parallel to the long axis of the myocyte.^7^ Valvular interstitial cells are fibroblast-like in nature with limited intercellular adhesions as they secrete and bind to their surrounding extracellular matrix (ECM) proteins.^8^ In response to cyclic stretching, their cytoskeleton is organized anisotropically along the principle stress direction.^3^ Vascular endothelial cells' cytoskeleton is generally aligned parallel to the shear stresses associated with blood flow and the tissues develop continuous layers with intercellular connections formed by tight junctions and adherens junctions.^9,10^ For these cardiovascular cells, the direct coupling of the cellular cytoskeleton to the ECM facilitates the ability of the network to maintain cell orientation and provides spatio-chemical cues that regulate organogenesis and function.

Cytoskeletal organization in cells cultured on micropatterned two-dimensional (2D) substrates has been reported in cardiomyocytes,^11–13^ valvular interstitial cells,^14^ and endothelial cells;^15,16^ however, the comparative architectures of these cells is not well studied in three dimensional (3D) scaffolds. Fiber spinning tissue scaffolds intends to recreate ECM-like polymer networks to support the growth and function of tissues.^17–20^ In these engineered tissues, fiber scaffolds are designed to mimic the ECM and provide structural cues at the nanometer and single micrometer scale, consistent with native ECM fiber diameters.^21^ These scaffolds have been used to create anisotropic heart valves,^19,20,22^ ventricles,^17,23^ and blood vessels.^24,25^

Here, we asked if cardiovascular cell types have different propensities for cytoskeletal alignment on 3D fiber scaffolds. Differences in cell types' 3D alignment tendencies would suggest that engineered tissue alignment is cell type dependent. Similarities would suggest common laws across heart cell types for the way they process geometric cues in the extracellular environment. To address this question, we seeded cells, with a constant density, onto fibrous scaffolds with controlled alignment and quantified actin anisotropy on the different fiber conditions. The fibers were fabricated with biocompatible poly(caprolactone) (PCL)/gelatin using focused rotary jet spinning (FRJS).^17^ We compared the actin networks of three cell types that are representative of major cardiovascular components: neonatal rat ventricular myocytes (NRVMs), ovine valvular interstitial cells (VICs), and human umbilical vein endothelial cells (HUVECs). The results presented show that the VIC actin networks more readily aligned on the moderately anisotropic scaffold conditions than the NRVMs' and HUVECs' actin. However, the three cell types showed similar alignment of their intracellular architectures after attachment to the highly aligned fiber scaffolds. These data suggest that cardiovascular cell types respond to external 3D boundary conditions with similar processes for organizing their inter- and intracellular architectures.

## RESULTS AND DISCUSSION

### Controlling fiber scaffold anisotropy

**FIG. 1.**
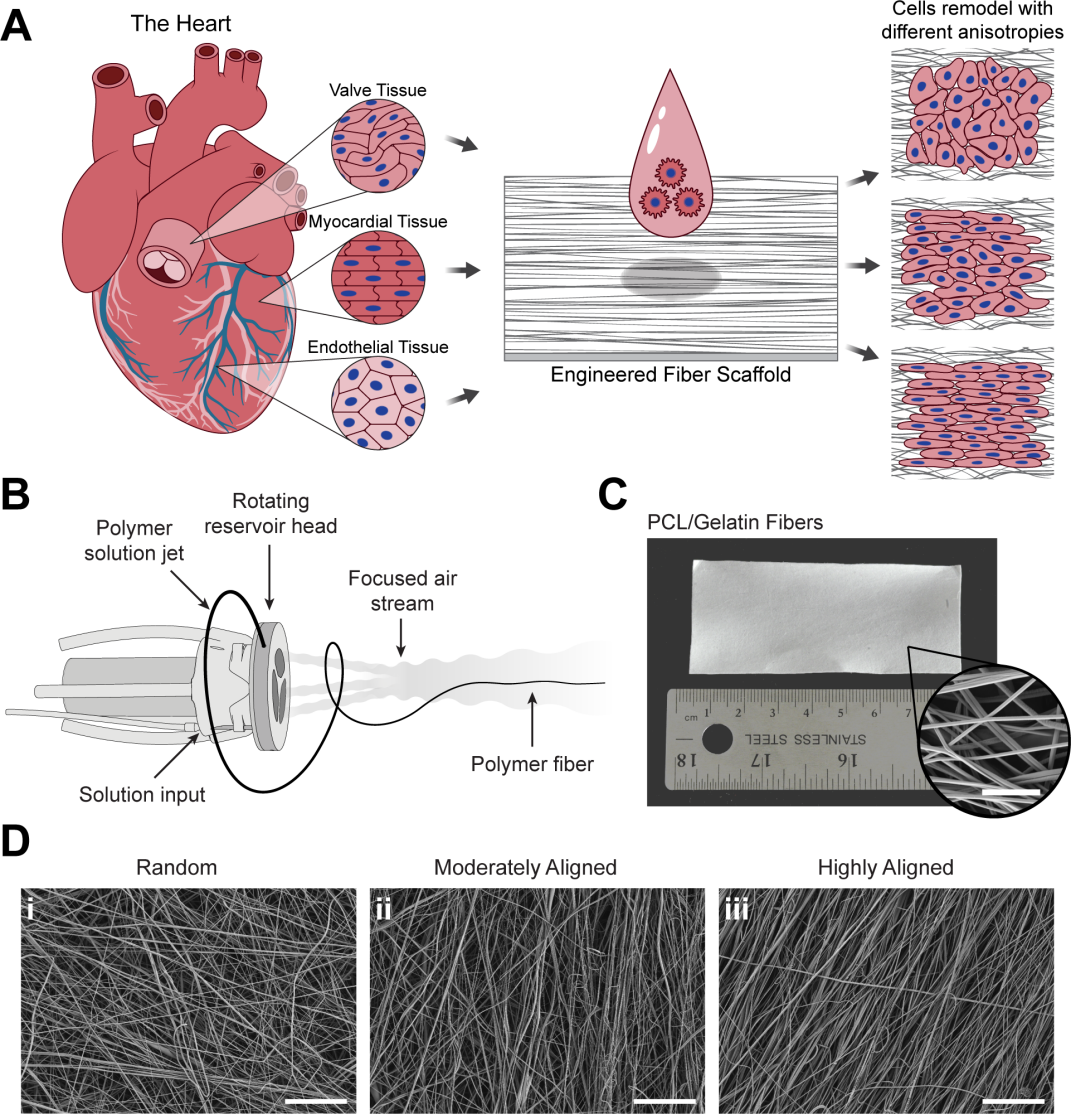
Cardiovascular cell types and engineered fiber scaffolds. (a) The heart is made up of a variety of anisotropic tissues, including the valve, myocardium, and endothelium. After seeding these different cell types onto engineered fiber scaffolds, the cells reorganize to form tissues with varying levels of anisotropy. (b) The FRJS creates a polymeric fiber stream by extruding a polymer solution through small orifices in a rotating reservoir. The extruded solutions form polymer jets that result in fibers which are directed and focused by a compressed air stream positioned near the rotating reservoir. (c) As a result, large sheets composed of micro- and nanofibers are produced (scale bar = 10 *μ*m). (d) These fiber sheets can be organized in different configurations, including (i) random, (ii) moderately aligned, and (iii) highly aligned (scale bars = 100 *μ*m).

**FIG. 2.**
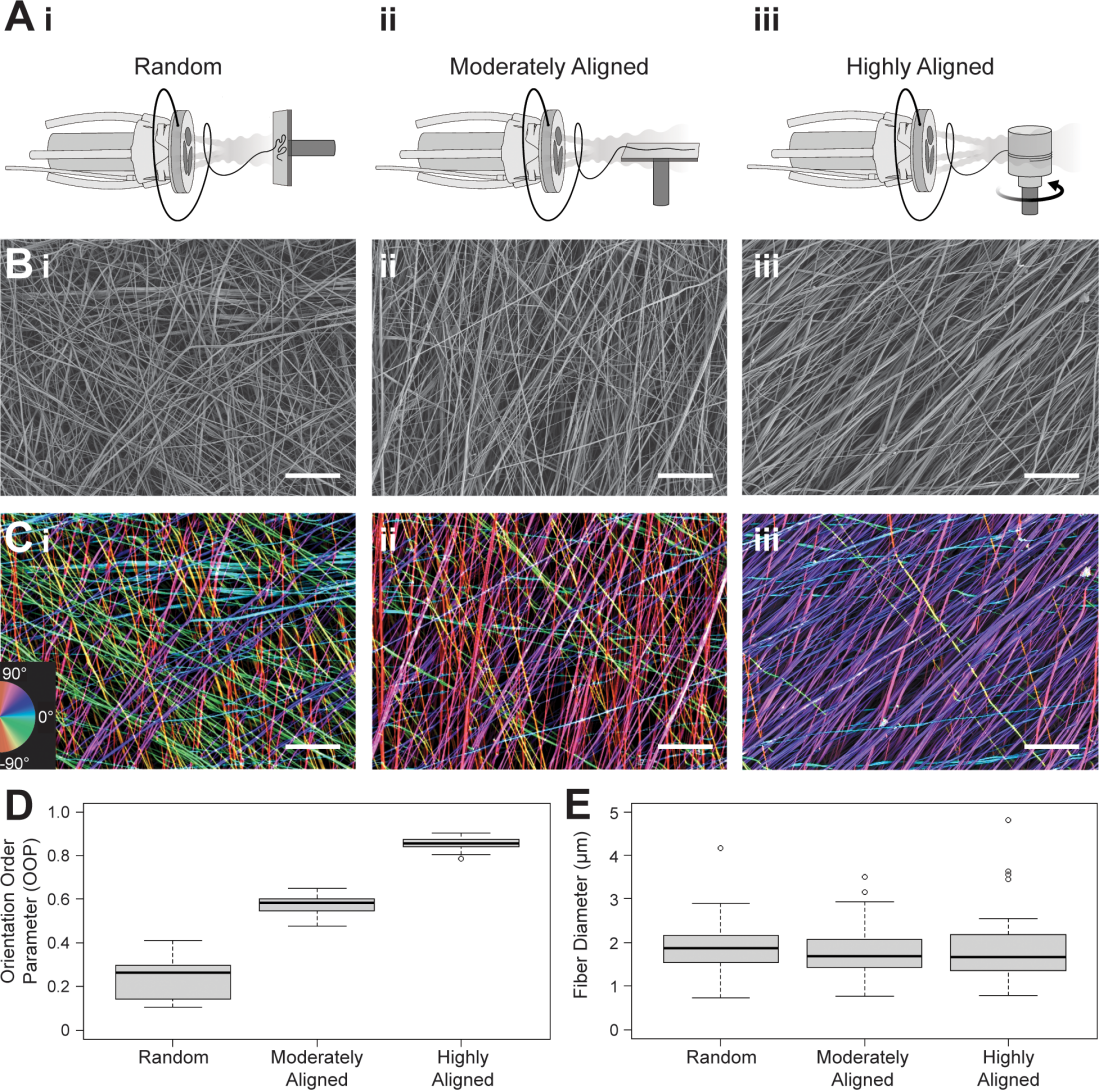
Quantifying fiber alignment. (a) By changing the angle and rotation of the collector, the fiber scaffold's organization can be controlled to produce (i) random, (ii) moderately aligned, and (iii) highly aligned substrates. (b-i)–(b-iii) The SEM images show the scaffold arrangement for the three scaffold organizations (scale bars = 100 *μ*m). (c-i)–(c-iii) The images were colorized based on fiber angle, resulting in a visualization of their alignment before their (d) alignment was quantified using OOP, showing the differences in anisotropy between the three conditions. (N = 9 samples across 3 production runs, box and whisker plots represent quartile values) (e) There was not, however, a difference in the distribution of fiber diameter in the three conditions. N = ∼150 fibers across 3 production runs; box and whisker plots represent quartile values.

To compare the adaptation of myocardial, valve interstitial, and endothelial cells to 3D environments, we seeded the cells on fiber scaffolds with differing alignments [Fig. 1(a)]. These ECM-mimicking biofiber scaffolds were produced using FRJS, a high throughput fiber manufacturing platform capable of controlling scaffold anisotropy.^17,26^ To encourage cell attachment and cell survival on the scaffolds, PCL/gelatin fibers were chosen as they have shown reliable biocompatibility and the ability to promote cell adhesion.^23,27,28^ A PCL/gelatin solution was injected into a rotating reservoir which utilizes centrifugal forces to radially expel polymeric fiber jets [Figs. 1(b) and 1(c)]. These radially extruded fibers are then directed by a pressurized air stream that focuses them onto a nearby collector. During spinning, the focused air creates an aligned stream of fibers that can then be arranged in random, moderately aligned, or highly aligned configurations by changing the orientation (with respect to the focused air stream) and rotation of the collection mandrel [Fig. 1(d)]. Orienting the collector perpendicular with respect to the fiber stream yielded fibers in a random (isotropic) configuration [Fig. 2(a-i)]. Adjusting the collector angle to be parallel with the fiber stream produced moderately aligned (moderately anisotropic) fiber scaffolds [Fig. 2(a-ii)]. Rotating the collection mandrel at 5000 RPM induced a highly aligned (highly anisotropic) configuration [Fig. 2(a-iii)]. Controlling the fiber anisotropy by altering the collection method allows for different scaffold organizations without affecting fiber formation and diameter.

Upon completion of the spinning procedure, fiber sheets were removed from the collector and prepared for testing. To confirm that these collection conditions resulted in distinct fiber organizations, fiber alignments were quantified using scanning electron microscopy (SEM) [Fig. 2(b)]. The scaffolds' orientation order parameter (OOP) was used as a metric for overall fiber alignment on a normalized scale from 0 to 1 [Figs. 2(c) and 2(d)]. An OOP of 0 represents a completely random fiber configuration, while an OOP of 1 represents a perfectly aligned fiber arrangement. The random fiber scaffold condition yielded an OOP of 0.24 ± 0.09, the moderately aligned condition produced an OOP of 0.58 ± 0.05, and the highly aligned condition displayed an OOP of 0.85 ± 0.03 [Fig. 2(d)] (values reported as mean ± standard deviation). These three values provide distinct OOPs that span the normalized range and allow for observation regarding different cardiovascular cell types' (cardiac muscle, valve, and endothelial cells) propensity for alignment on differing levels of 3D scaffold anisotropy.

As cellular response and organization can be affected by fiber diameter,^29,30^ homogenous fiber formation represents an important fabrication parameter. To ensure that our fiber collection processes did not affect fiber size, we imaged the scaffolds and quantified the fiber diameter across collection conditions. Here, there were no significant differences between the fiber scaffold groups with an average fiber diameter between 1.5 and 2 *μ*m and a range spanning between the hundreds of nanometer to single micrometer range [Fig. 2(e)]. These fiber diameters are similar to fibrous collagen I and other abundant ECM proteins,^31^ meaning the scaffolds represent spatial scales that these cell types interact with in the body.

### Fiber scaffold mechanical anisotropy

**FIG. 3.**
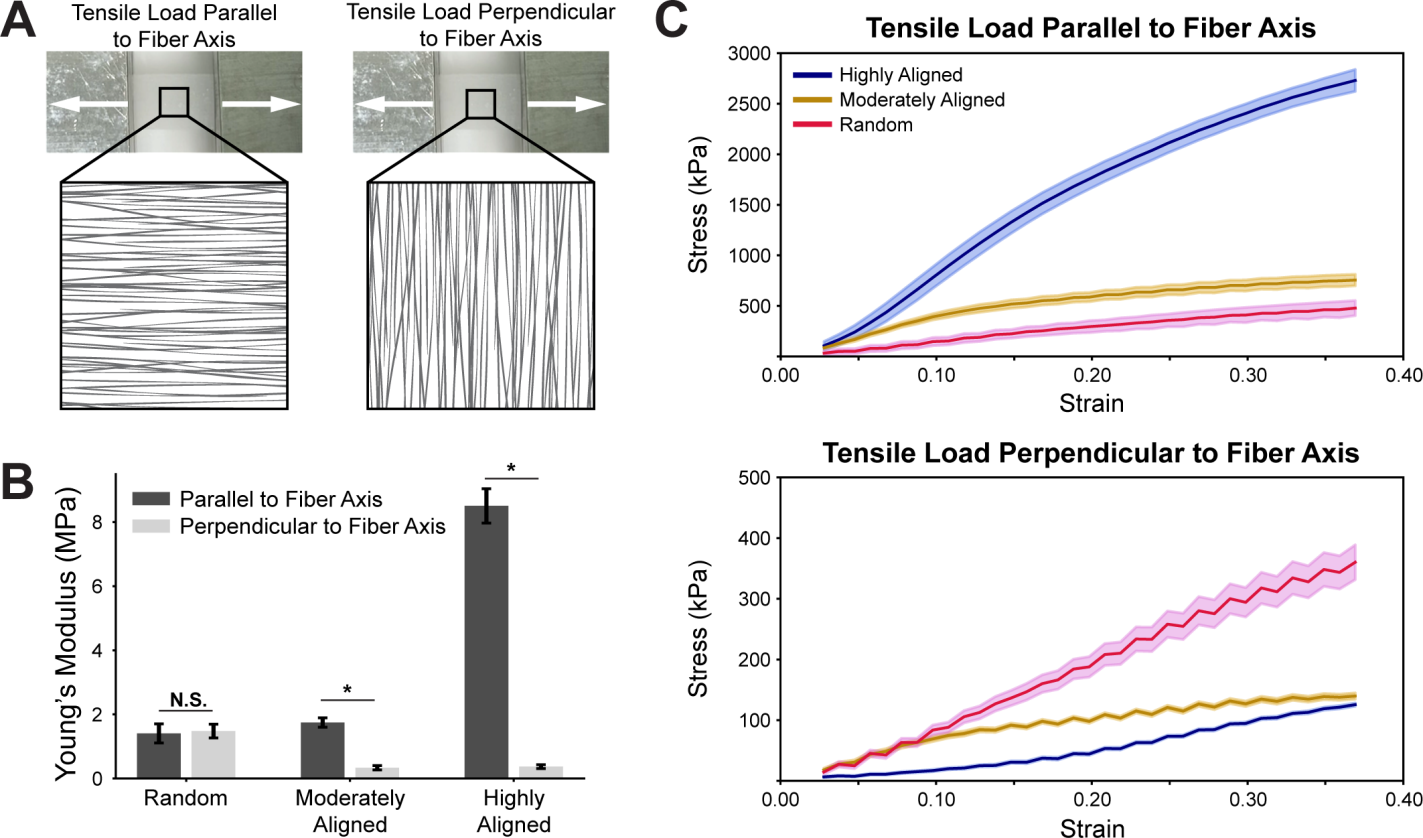
Fiber scaffolds demonstrate mechanical anisotropy. (a) PCL/gelatin fiber scaffolds were tested along two axes: parallel and perpendicular to the fiber alignment. (b) Young's moduli were calculated from the tensile tests for all three scaffold conditions. (c) Stress vs strain curves show differences in scaffold behavior dependent on the load direction of the tensile test. N = 5 for all conditions; line represents average stress and error indicates standard error of the mean; * indicates p < 0.05.

To confirm mechanical anisotropy of the fiber scaffolds, we performed biaxial tensile tests parallel and perpendicular to the axis of fiber alignment. Prior to tensile testing, fiber scaffolds were cut into 0.5 cm wide strips with strips cut in two directions: (1) along the fiber axis and (2) perpendicular to the fiber axis [Fig. 3(a)]. Generally, anisotropic fiber scaffolds show higher tensile strength along the axis of alignment as the forces pull more directly along the fibers as opposed to simply pulling them apart laterally.^32,33^ Isotropic fiber scaffolds, however, do not have a dominant alignment direction and do not exhibit any directional differences in mechanical stiffness. Biaxial mechanical testing revealed no significant difference in the Young's moduli for the random fiber scaffolds; however, there was a significant difference between the Young's moduli of the two pulling directions in the moderately aligned and highly aligned scaffolds [Fig. 3(b) and Table S1]. The moderately aligned scaffolds displayed a ∼4.7 times higher Young's modulus when tested along the fiber alignment direction compared to perpendicular to the fiber alignment direction. Similarly, the highly aligned scaffolds displayed ∼25 times higher Young's modulus along the fiber alignment axis. Altogether, these results confirm both the isotropy of the random condition and the increasing anisotropy of the aligned scaffolds since we expect differences in Young's modulus to increase as anisotropy increases.

### Fiber alignment direction potentiates cytoskeletal alignment direction

**FIG. 4.**
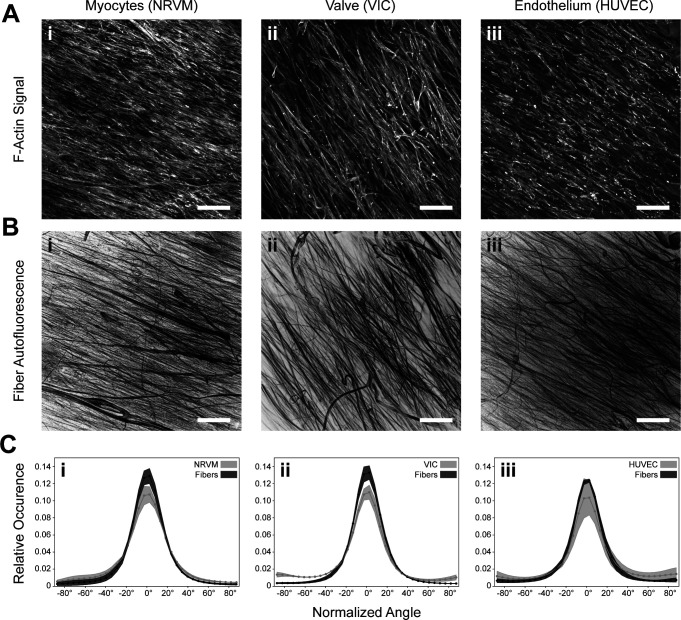
Cytoskeletal alignment direction is dictated by the fiber scaffold. (a-i)–(a-iii) In the highly aligned fiber condition, cytoskeletal networks, stained for F-Actin, and (b-i)–(b-iii) fiber scaffold displayed similar angular alignment. (c-i)–(c-iii) Plots normalized to the dominant fiber angle display that the distribution of these alignments match in all three cell types. N = 3 samples across 3 experimental runs; error represents standard deviation; all scale bars are 200 *μ*m; samples were fixed and imaged after 5 days in culture.

To evaluate the intracellular architecture and general cell alignment, we used actin networks as an indicator for cytoskeletal shape. In efforts to determine the relationship between fiber and cytoskeletal alignment directions, we seeded cells onto highly aligned fibers and compared cells' actin networks to the underlying scaffolds. The cells used in this study included neonatal rat ventricular myocytes (NRVM), ovine valvular interstitial cells (VIC), and human umbilical vein endothelial cells (HUVEC). Cell types were chosen on the basis of their use in animal or *in vitro* models: namely, NRVMs for myocardial structural morphology and functionality,^12,34^ ovine VICs for valve tissue formation and regeneration,^8,35^ and HUVECs for general vascular models.^36^ All fiber scaffolds were seeded at 1M cells/cm^2^. Different seeding concentrations could influence the cells' alignment behaviors as crowded or sparse intercellular environments might induce different cellular responses.^37^ The actin network was stained to visualize the angular distribution of the cells, while the scaffolds' auto fluorescent properties when exposed to a 640 nm laser were used to visualize the fibers. By imaging the fibers concurrently with the cells, but in different optical channels, we were able to visualize both components in the same field of view, allowing for comparisons of angular distributions [Figs. 4(a) and 4(b)]. In all three cardiovascular cell types, the cytoskeletal angular distribution correlated with the fibers' angular distribution [Figs. 4(c)]. These data suggest that local anisotropic boundary conditions whose width scale are on the order of one micrometer are sufficient to potentiate the alignment of cells in the heart's musculature, valves, and vasculature.

### Nuclear morphology on fiber scaffolds

**FIG. 5.**
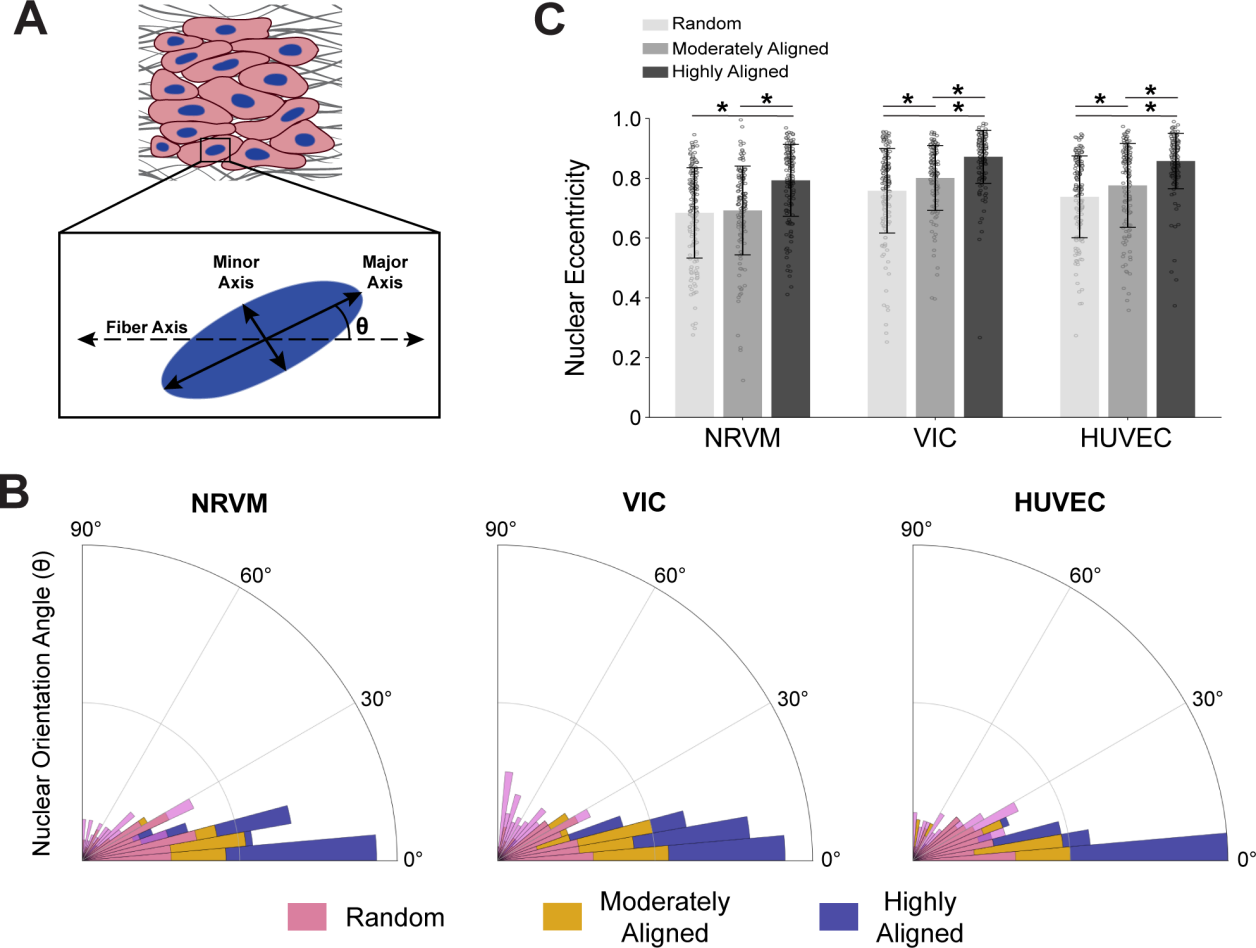
Nuclear orientation angle and eccentricity on fiber scaffolds. (a) Nuclei tend to elongate themselves into an elliptical shape with a major and minor axis that is offset from the fiber axis by an angle θ (nuclear orientation angle). (b) The nuclear orientation angle was plotted for each cell type and scaffold condition. Histogram values represent the absolute value of the angular difference. The distribution of nuclear angles became more aligned with the fibers (0°) as the scaffold anisotropy increased for all three cell types. (c) Nuclear eccentricity for the three cell types on the three scaffold conditions shows the nuclei tended to elongate as the fiber scaffolds became more aligned. For all data represented N = ∼150 nuclei across 3 biological replicates/condition; bar plots represent mean ± standard deviation and * indicates p < 0.05; samples were fixed and imaged after 5 days in culture.

Cytoskeletal alignment is also reflected in nuclear morphology as the cytoskeleton is the mechanical link between the nucleus and the cell membrane. Extracellular mechanical forces, transmitted to the nucleus via ECM-integrin binding through the cytoskeleton, can regulate important nuclear functions and characteristics, including chromatin structure and gene expression.^38–40^ Nuclear shape and orientation indicate the magnitude and direction of stress fields within the cell as the cytoskeletal organization tends to lengthen the nucleus into an ellipsoid with the major axis aligning along the cell's stress field^41–43^ [Fig. 5(a)].

In this study, the cell's cytoskeletal organization aligned with the fiber scaffold's direction (Fig. 4). We expected to see the nuclei align and elongate similarly as the anisotropy of the fiber scaffolds increased. To show this relationship, we quantified nuclear orientation angle with respect to the dominant fiber axis [Fig. 5(b)]. We defined the nuclear orientation angle (θ) as the absolute value of the angle difference between the nucleus's major axis and the dominant fiber axis [Fig. 5(a)]. In all cell types and scaffold conditions, θ was centered around 0° (fiber scaffold's dominant axis) [Fig. 5(b) and Table S2]. The distribution of nuclear orientation angles narrowed as the scaffold alignment increased for all cell types [Fig. 5(b)]. Cells seeded on moderately aligned scaffolds had a smaller nuclear orientation angle deviation from the average than the random scaffolds; however, the cells seeded on the highly aligned scaffolds still displayed the narrowest angle distribution of the three fiber conditions. The nuclear orientation angle results reinforce the notion that there are increased cytoskeletal alignments along the scaffold axes as the actin networks are responsible for orienting the nuclei.

In addition to the angle, the extent to which the nuclei are elongated also provides some insight into the intracellular stresses. Cytoskeletal stresses can pull the nuclear membrane, creating an ellipsoid with increasing length difference in the major and minor axes [Fig. 5(a)]. We quantified the nuclear eccentricity as a normalized measurement of the relationship between the major and minor axes [Fig. 5(c)]. All three cell types showed an increasing trend in nuclear eccentricity as the fiber scaffold anisotropy increased, signifying larger levels of intracellular stress transferred to the nuclei. The NRVMs displayed lower nuclear eccentricities than both the VICs and the HUVECs; however, this is consistent with NRVM,^41^ VIC,^14^ and HUVEC^16^ cultures on 2D micropatterned substrates where the cardiac muscle cell nuclei did not elongate as much as the other two cell types. Cardiomyocytes have been shown to form circumnuclear microtubule organization centers (MTOC) as opposed to proliferative cells' development of centrosomal MTOCs.^44^ The non-centrosomal MTOC of the cardiomyocytes has been suggested to be mechanically advantageous in terms of contractile forces, cell stability, and sarcomere organization.^45^ A more distributed MTOC in the cardiomyocytes might explain the lower nuclear eccentricity of the NRVM as the centrosomal MTOCs (present in the VICs^46^ and HUVECs^47^) polarize the cell, enabling higher intracellular stresses and nuclear elongation.^40^ While the nuclear morphology of the three cell types seeded upon engineered fiber scaffolds is consistent with MTOC formation, they do not yield immediate conclusions. More work is needed to evaluate downstream gene expression differences in the various anisotropy conditions.

### Cellular alignment on fiber scaffolds

To study each cell types' propensity for intracellular anisotropic remodeling relative to their local microenvironment, we cultured them on the three different fiber scaffold conditions (random, moderately aligned, and highly aligned), controlling for total cell population (1M cells/cm^2^). The cells were cultured for five days *in vitro* on fibronectin coated PCL/gelatin fibers, allowing the cells to remodel and organize relative to the scaffold. Including the fibronectin coating on the fiber scaffolds provided focal adhesion binding sites for all three cell populations. The integrin binding allows for extracellular mechanical and spatial cues to transfer across the cell membrane and potentiate intracellular reorganization. All three cell populations express α_5_β_1_ integrin at relatively high levels.^48–50^ The α_5_β_1_ integrin heterodimer is a major fibronectin receptor and responsible for the preferential binding to fibronectin for all three cell populations.^51^

**FIG. 6.**
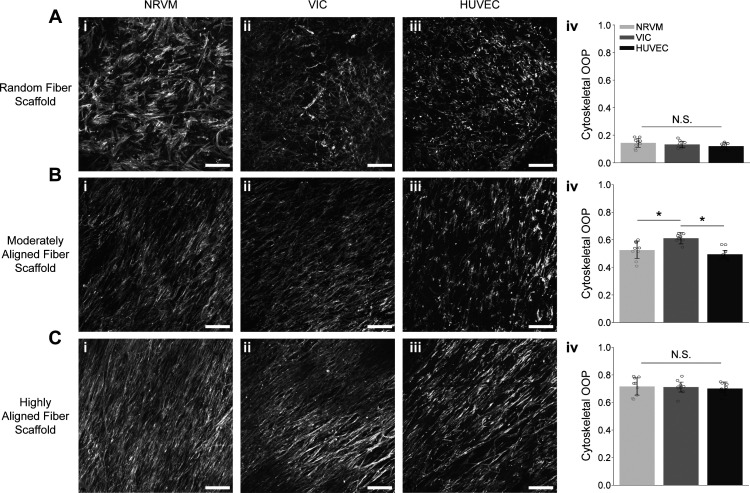
Cardiovascular cells' actin alignment on fiber scaffolds. Samples use a phalloidin stain to visualize the F-actin filaments, representing the cytoskeletal organization. (a) When seeded onto randomly aligned scaffolds, (i) NRVMs, (ii) VICs, (iii) and HUVECs displayed isotropic actin networks. (iv) There was no statistically significant difference between the actin organization in the three cell types on the random fiber scaffolds. (b) On the moderately aligned fiber scaffolds, (i) NRVMs, (ii) VICs, and (iii) HUVECs showed an intermediate degree of cytoskeletal alignment, organizing their actin networks parallel to each other. (iv) The VIC cytoskeleton showed a higher cytoskeletal OOP than both HUVEC and NRVM. (c) (i) NRVMs, (ii) VICs, (iii) and HUVECs seeded on the highly aligned fiber scaffolds showed the largest amount of cytoskeletal anisotropy, configuring themselves in a highly parallel manner, creating an aligned tissue. (iv) Under the highly aligned condition, there was no statistically significant difference between the actin organization in the three cell types. N = 9 samples across 3 experimental runs; bar plots represent mean ± standard deviation and * indicates p < 0.05; all scale bars are 200 *μ*m; samples were fixed and imaged after 5 days in culture.

After five days, the cells were fixed and stained to visualize the nuclei and cytoskeletons. We then imaged the cells and used the cytoskeletal signal to measure the actin OOP for each condition (Fig. 6). The actin OOP values were calculated using multiple fields of view, each measuring over 1 mm^2^ to show the bulk collective population behavior as opposed to local organizations. Statistical comparisons were made between cell types on the same fiber scaffolds to evaluate the cells' relative propensity for alignment.

We expected the cells' actin OOP to increase as the underlying scaffolds' anisotropy rose. This relationship held true in all cell types; however, the magnitude of increase for cytoskeletal OOP varied across the three populations. The NRVMs, VICs, and HUVECs displayed similar organizational behaviors in the two extreme cases of random and highly aligned fibrous scaffolds. Under the random condition, the actin OOP values were 0.14 ± 0.03 for the NRVM, 0.13 ± 0.02 for the VIC and 0.12 ± 0.02 for the HUVEC (OOP values reported as mean ± standard deviation) [Fig. 6(a-iv)]. Additionally, under the highly aligned condition, the actin OOP values were 0.72 ± 0.06 for the NRVM, 0.71 ± 0.05 for the VIC, and 0.70 ± 0.04 for the HUVEC [Fig. 6(c-iv)]. The consistent OOP values under the extreme conditions (random and highly aligned) of this study suggest that at both high and low levels of fiber scaffold organizations, the intracellular anisotropy is dictated by scaffold architecture and not cell type. Similar responses on the two extreme scaffold organizations could potentially result from consistent integrin–ECM interactions across the three cell populations, yielding fiber scaffold driven cytoskeletal organizations.

Under the moderately aligned conditions, however, the endothelial and myocardial tissues differed from the valve cell population in their cytoskeletal alignment. The VICs showed a higher tendency toward alignment, displaying an actin OOP of 0.61 ± 0.03, higher than both the NRVM (0.53 ± 0.06) and the HUVEC (0.49 ± 0.04) [Fig. 6(b-iv)]. These results indicate that VICs have a higher propensity for cytoskeletal anisotropy on scaffolds with an intermediate alignment in the current experimental design. These data suggest a lower alignment threshold among VICs compared to NRVMs and HUVECs. Human VICs have been shown to differ from NRVM and HUVEC in their relatively low expression of cadherins and connexins,^52^ common cell-cell coupling molecules. Higher levels of lateral cell coupling in both NRVMs and HUVECs could potentially limit their degree of intracellular anisotropy, potentially explaining the lower cell alignments compared to the VICs.

Others have quantitatively evaluated cardiovascular cell anisotropy on random and aligned fibers, studying the cells' ability to self-organize on the 3D scaffolds. Mancino *et al.* reported anisotropic polypyrrole fiber scaffolds potentiated alignment in NRVM cultures after 7 days using a related metric, coefficient of alignment.^53^ However, they did not report a large alignment discrepancy in their cell populations seeded on aligned and nonaligned fiber scaffolds. Additionally, Kai *et al.* achieved rabbit cardiomyocyte alignment control on anisotropic PCL/gelatin fibers, similar in material composition to our study, but reported their findings through qualitative SEM images.^54^ Previous results in VIC cultures on fiber scaffolds also found alignment changes when seeding onto random and aligned scaffolds; Masoumi *et al.* showed VICs ability to align along anisotropic fiber scaffolds and inability to align when seeded on isotropic scaffolds.^55^ Their results focused on the quantification of nuclei directionality as a proxy for cytoskeletal behavior. Finally, Whited and Rylander reported increasing alignment of HUVECs as their underlying PCL/collagen fiber scaffold anisotropy increased, quantifying cell alignment by examining the average cell angle deviation from the main fiber axis.^56^ In all, these results corroborate the three cell lines' behavior in this study. However, comparative analyses between cardiovascular cells' cytoskeletal organization were not previously effective due to cross-study differences in fiber scaffold properties (e.g., fiber type, degree of alignment, and fiber diameter) and cell alignment quantification methods. By standardizing both fiber scaffolds and the alignment quantification technique, we were able to directly compare the intracellular organization behaviors of major cardiovascular cell types.

## CONCLUSIONS

We asked whether cardiovascular cell types have different cytoskeletal alignment propensities when cultured on 3D fibrous scaffolds. To answer this question, we fabricated fiber scaffolds with controlled alignments and measured myocardial, valve, and endothelial cells' response to the manufactured extracellular environment. The cardiovascular cell types we used behaved similarly, remodeling their cytoskeleton relative to the geometric cues embedded within the fibrous scaffold. This is important because it suggests that integrin binding of the ECM triggers common cytoskeletal organization. Thus, this result would imply that differences in the cellular microenvironment of the ventricular myocardium, valve leaflets, and vasculature are dependent on subtle, localized differences in ECM, cell populations, applied mechanical stresses, diffusion constants of locally produced signaling molecules, and the gene expression profile, specifically the integrin expression, of the cell type of interest. For the tissue engineer, this suggests granular design requirements that may exist at spatial scales smaller than what current additive manufacturing techniques can produce.

## METHODS

### Fiber scaffold fabrication

Poly(ε-caprolactone) (PCL) (Sigma Aldrich, St. Louis, MO, USA) and pure gelatin (from Porcine skin, gel strength 300, Type A; Sigma Aldrich, St. Louis, MO, USA) were dissolved in 1,1,1,3,3,3-Hexafluoro-2-propanol (HFIP) (Oakwood Chemical, Estill, SC, USA) at 4% (w/v) and 2% w/v, respectively. The solution was spun using focused rotary jet spinning (FRJS).^17,26^ PCL/gelatin solution was loaded into a 60 ml syringe and fed into a rotating reservoir in the FRJS system at a rate of 1 ml per minute. The aluminum reservoir was oriented perpendicular to the air stream and rotated at 10 000 RPM. The focused air stream pressure was kept constant at 0.2 MPa. Each fiber scaffold was generated using 15 ml of solution. Random fibers were spun onto a non-rotating rectangular mandrel coated with MR311 Dry Film Release Agent (Sprayon, Cleveland, OH) and oriented perpendicular to the fiber stream. The moderately aligned fibers were spun onto a flat, rectangular mandrel coated with MR311 Dry Film Release Agent and oriented parallel to the fiber stream. To generate highly aligned fibers, fibers were spun onto a cylindrical mandrel coated with MR311 Dry Film Release Agent and rotating at 5000 rpm.

### Scanning electron microscopy

To prepare the fibers, scaffolds were cut into 5 × 5 mm^2^ square samples for scanning electron microscopy (SEM). The samples were secured to metal stubs using carbon tape. A MS300T D Dual Head Sputter Coater was used to coat 10 nm Pt/Pd 80/20 onto the sample prior to imaging. A Zeiss Supra field-emission scanning electron microscope (FESEM) at 5 kV with an SE2 detector imaged the samples to create micrographs. The images were taken at 500× magnification to view the general scaffold organization.

### Mechanical testing of scaffolds

Random, moderately aligned, and highly aligned scaffold samples were sectioned into 5 ×30 mm^2^ pieces. The individual samples obtained from the scaffolds were oriented either parallel or perpendicular to the fiber axis in the anisotropic conditions. For the random condition, fiber samples were sectioned in strips perpendicular to each other to test the material biaxially. A digital caliper (Mitutoyo Absolute Digimatic, Japan) was used to measure sample thickness. Samples were tested on a CellScale Biaxial Tester (2.5N load cells, CellScale, Ontario, Canada) submerged under water at 37 °C with a gauge length of 10 mm. Samples were tested at a strain rate of 1% per second.

### Fiber orientation analysis

Using the SEM images, the orientation order parameter (OOP) was determined to compare fiber alignment of random, moderately aligned, and highly aligned fiber scaffolds. ImageJ with the OrientationJ plugin (Biomedical Image Group, EPFL, Switzerland) was used to analyze the alignment of the scaffold fibers by creating a color-mapped image of the scaffold based on the angle of individual fibers.^57^ This angle of the individual fibers was found by using foreground pixels and assigning the orientation of the local neighborhood using a structure tensor method (OrientationJ plugin). These pixel-by-pixel angle measurements create the color-mapped image. The total set of color values then represents a distribution for the total set of fiber angles. Following OrientationJ processing, the OOP was calculated using a custom MATLAB code, as previously described,^58^ by assigning a normalized value (between 0 and 1) based on the width of the angle's distribution (MATLAB and Statistics Toolbox Release 2018b, The MathWorks, Inc., Natick, MA, USA).

### Fiber diameter analysis

To analyze the fiber diameters, we quantified SEM images. The fiber diameters were measured using ImageJ's measurement tool (ImageJ v. 1.53e). Three separate fiber production runs were imaged and analyzed and >50 fiber diameters were measured from each production run.

### Fiber scaffold cell seeding preparation

In preparation for cell cultures, scaffolds were cut into 20 × 5 mm^2^ pieces. The ends of each scaffold were adhered to the bottom of a 12-well culture plate using clear nail polish (Electron Microscopy Sciences, Hatfield, PA, USA); nail polish was left to dry for at least 2 h. The wells were then filled with 70% ethanol left to evaporate overnight. The scaffolds were further treated under ultraviolet (UV) light for 30 min. After ethanol and UV sterilization treatments, the scaffolds were washed three times with Phosphate Buffer Saline (PBS) (Gibco, Thermo Fisher Scientific, USA) to remove any ethanol still present. Prior to seeding, the scaffolds were submerged in 10 *μ*g/ml fibronectin (human natural fibronectin, Corning, NY, USA) for 1 h. All three cell types were seeded at 1M cells/cm^2^ of fiber scaffold).

### Ventricular myocyte isolation and culture

Neonatal rat ventricular myocytes were isolated from two day old Sprague-Dawley rats using a previously published method.^59^ The protocol was reviewed and approved by the Institutional Animal Care and Use Committee (IACUC). Cells were cultured using Medium 199 (Gibco, ThermoFisher Scientific, USA) supplemented with 0.1 mM Minimum Essential Medium (MEM) nonessential amino acids, 10% heat-inactivated Fetal Bovine Serum (FBS), 10 mM HEPES, 3.5 g/l glucose, 2 mM L-glutamine, and 2 mg/l vitamin B12. After 2 days in culture, the media formulation was changed by lowering the FBS concentration to 2% while keeping other supplements consistent. All cultures were kept in sterile conditions at 37 °C and 5% CO_2_.

### Valvular interstitial cell isolation and culture

Valvular interstitial cells were isolated from pulmonary heart valve leaflets of adult sheep obtained from a slaughterhouse (Blood Farm, Groton, MA). Briefly, leaflet tissues were cut in small pieces and plated in petri dishes with a collagenase solution for 10 min to remove valvular endothelial cells. Valvular interstitial cells were then isolated by placing the leaflets in a separate collagenase solution for 2 h at 37 °C for full leaflet digestion. The resulting digestate was centrifuged at 250 g for 5 min and resuspended and cultured in Advanced Dulbecco's modified Eagle medium (Sigma Aldrich, St. Louis, MO, USA), supplemented with 10% FBS (Invitrogen, Waltham, MA, USA), 1% Glutamax (Invitrogen, Waltham, MA, USA), and 1% Penicillin-Streptomycin (Invitrogen, Waltham, MA, USA), at 37 °C and 5% CO_2_.

### Endothelial cell culture

Human umbilical vein endothelial cells (Lonza, Basel, Switzerland) were used for the endothelial cell cultures in this study. Cells were grown and maintained using Medium 200 (Gibco, MA, USA) supplemented with 2% low serum growth supplement (Thermo Fisher Scientific, MA, USA) and 1% Penicillin-Streptomycin (Invitrogen, Waltham, MA, USA). All cultures were kept under sterile conditions in a cell incubator at 37 °C and 5% CO_2_.

### Immunofluorescent staining

Seeded scaffolds were rinsed three times with 37 °C PBS (Gibco, Thermo Fisher Scientific, MA, USA) within a 12 well culture plate and then fixed with 4% Paraformaldehyde (Electron Microscopy Sciences, Hatfield, PA, USA) for 10 min. After fixing, scaffolds were rinsed in PBS three times. All PBS was aspirated, and cells underwent permeabilization with 2 ml of 0.2% Triton-X (Sigma Aldrich, St. Louis, MO, USA) in PBS for 10 min at room temperature. Scaffolds were then rinsed three times with PBS. After permeabilization, the samples were blocked with 2 ml of 5% v/v Bovine Serum Albumin (BSA; Sigma Aldrich, Saint Louis, MO, USA) added to each well and incubated at room temperature for 15 min. Scaffolds were again rinsed three times with PBS. The cell-seeded scaffolds were cut into small 5 × 5 mm^2^ squares and transferred to a petri dish, where they were ready for staining.

The cardiomyocyte samples were incubated in 1:200 dilutions of mouse anti-sarcomeric *α*-actinin (Sigma Aldrich, St. Louis, MO, USA) for 2 h. After primary incubation, samples were rinsed three times in PBS. All samples were then incubated at room temperature for 2 h in a solution containing 1:250 dilution Phalloidin conjugated to Alexa-Fluor 488 (Invitrogen, Waltham, MA, USA) and 1:250 dilution 4′,6-diamidino-2-phenylindole (DAPI) (Sigma Aldrich, St. Louis, MO, USA). The cardiomyocyte samples were also incubated in a secondary antibody solution containing goat anti-rabbit IgG (H + L) conjugated to Alexa-Fluor 546 (1:250 concentration in PBS; Life Technologies; Carlsbad, CA, USA). The samples were covered with aluminum foil while staining to protect from photo bleaching. Following secondary incubation, the samples were washed in PBS three times and stored in a 4 °C refrigerator in PBS.

### Confocal imaging

A spinning disk confocal microscope (Olympus ix83, Andor spinning disk) was used to acquire immunofluorescent images at wavelengths of 405, 488, 561, and 640 nm wavelengths. Regions of interest were imaged at 10× and 20× magnification. Images were captured using *z*-stacks to capture the 3D nature of the cell and fiber scaffolds. The *z*-stacks spanned 5–20 *μ*m of scaffold depth.

### Actin orientation analysis

Representative *z*-stacks in the 488 nm channel were converted to a single image using maximal intensity projection in ImageJ. These projections were then analyzed using OrientationJ, an ImageJ plugin (Biomedical Image Group, EPFL, Switzerland). Similar to fiber orientation analysis, the plugin generated a color-mapped image based on the angle of each actin filament. Each color-mapped image was imported to MATLAB (MATLAB and Statistics Toolbox Release 2018b, The MathWorks, Inc., Natick, Massachusetts, United States) and a previously described custom MATLAB code^58^ was run to calculate the orientation order parameter (OOP) of each image.

### Fiber and cell angular distribution

Maximal intensity projections were produced for the 488 nm channel (Phalloidin stain) and the 640 nm (PCL/gelatin fiber autofluorescence) in the same region of interest. Each image was processed in ImageJ using OrientationJ's Distribution analysis function which outputs a distribution of angular occurrences from −90° to 90°. These distributions were then compiled for each cell type and the underlying fibers for those cell types and plotted on top each other.

### Nuclear morphology analysis

Nuclei images were produced using maximal intensity projections from the 405 nm channel (DAPI stain). These images were processed in ImageJ. Before analysis, images were normalized to a 0° horizontal angle using OrientationJ's Horizontal Alignment function. The nuclei were then isolated using a thresholding feature and outlined with ImageJ's particle analyzer function. Major and minor axes as well as the nuclear orientation angle (θ) were quantified with ImageJ's measurement tool based on the nuclear outlines. The nuclear orientation angles were represented from 0° to 90° based on the absolute value of θ. Nuclear eccentricity (*e*) was calculated using

$$e=\sqrt{1-\frac{b^{2}}{a^{2}}},$$ (1)

where *a* represents the major axis length and *b* represents the minor axis length [Fig. 5(a)].

### Statistical analyses

For statistical group comparisons, analyses were carried out using Python (v2.1.1-Anaconda 3.9.7–64 bit). All groups were checked for normality using a Shapiro–Wilk test. Groups deemed as normal distributions were compared using a one-way ANOVA followed by a Tukey *post hoc* test. Groups that were not found to be normal distributions were compared using Kruskal–Wallis test (ANOVA on ranks) followed by a Mann–Whitney *post hoc* test. In all statistical analyses, p values below 0.05 were considered statistically significant. Bar plots were produced using the mean with the error bar representing standard deviation unless otherwise noted. All datasets were plotted using Python.

## SUPPLEMENTARY MATERIAL

See the supplementary material for additional information mechanical properties of fibers scaffolds and nuclear angle distributions.

## Data Availability

The data that support the findings of this study are available from the corresponding authors upon reasonable request.
